# Metabolomic Profiles Predict Diabetes Remission after Bariatric Surgery

**DOI:** 10.3390/jcm9123897

**Published:** 2020-12-01

**Authors:** Jane Ha, Mi Jang, Yeong-Keun Kwon, Young-Suk Park, Do-Joong Park, Joo-Ho Lee, Hyuk-Joon Lee, Tae-Kyung Ha, Yong-Jin Kim, Sang-Moon Han, Sang-Uk Han, Yoon-Seok Heo, Sung-Soo Park

**Affiliations:** 1 Department of Medicine, Korea University College of Medicine, Seoul 02841, Korea; janehapti@korea.ac.kr; 2 Department of Biotechnology and Food Science, Norwegian University of Science and Technology, 7491 Trondheim, Norway; kia3111@gmail.com; 3 Division of Foregut Surgery, Korea University College of Medicine, Seoul 02841, Korea; kukwon@korea.ac.kr; 4 Department of Surgery, Seoul National University Bundang Hospital, Seongnam 13620, Korea; youngsukmd@gmail.com; 5 Department of Surgery, Seoul National University Hospital, Seoul 03080, Korea; dojoongpark@gmail.com (D.J.P.); appe98@snu.ac.kr (H.-J.L.); 6 Department of Surgery, Nowon Eulji Medical Center, Seoul 01830, Korea; gsljh7@gmail.com; 7 Department of Surgery, Hanyang University College of Medicine, Seoul 04763, Korea; missurgeon@hanyang.ac.kr; 8 Department of Surgery, H+ Yangji Hospital, Seoul 08779, Korea; yjgs1997@gmail.com; 9 Department of Surgery, Cheil General Hospital, Seoul 04619, Korea; surgeryhan@gmail.com; 10 Department of Surgery, Ajou University Hospital, Suwon 16499, Korea; hansu@ajou.ac.kr; 11 Department of Surgery, Inha University Hospital, Incheon 22332, Korea; gshur@inha.ac.kr

**Keywords:** bariatric surgery, metabolic surgery, diabetes, amino acid, metabolomics

## Abstract

Background: Amino acid metabolites (AAMs) have been linked to glucose homeostasis and type 2 diabetes (T2D). We investigated whether (1) baseline AAMs predict T2D remission 12 months after bariatric surgery and (2) whether AAMs are superior for predicting T2D remission postoperatively compared with existing prediction models. Methods: Among 24 participants undergoing bariatric surgery, 16 diabetes-related AAMs were quantified at baseline and postoperative 3 and 12 months. Existing prediction models included the ABCD, DiaRem, and IMS models. Results: Baseline L-dihydroxyphenylalanine (L-DOPA) (areas under receiver operating characteristic curves (AUROC), 0.92; 95% confidence interval (CI), 0.75 to 1.00) and 3-hydroxyanthranilic acid (3-HAA) (AUROC, 0.85; 95% CI, 0.67 to 1.00) better predicted T2D remission 12 months postoperatively than the ABCD model (AUROC, 0.81; 95% CI, 0.54 to 1.00), which presented the highest AUROC value among the three models. The superior prognostic performance of L-DOPA (AUROC at 3 months, 0.97; 95% CI, 0.91 to 1.00) and 3-HAA (AUROC at 3 months, 0.86; 95% CI, 0.63 to 1.00) continued until 3 months postoperatively. Conclusions: The AAM profile predicts T2D remission after bariatric surgery more effectively than the existing prediction models.

## 1. Introduction

In recent years, it has become apparent that bariatric surgery not only promotes dramatic weight loss, but also induces the improvement or remission of type 2 diabetes (T2D) [1,2]. Current clinical guidelines recommend that bariatric surgery be considered to treat inadequately controlled T2D in people with a body mass index (BMI) as low as 30 kg/m^2^ or as low as 27.5 kg/m^2^ in Asian patients [3,4]. However, previous studies showed that 25% to 65% of T2D patients did not experience postoperative T2D remission [1,2] and that over 20% of patients with T2D remission experienced T2D recurrence during long-term follow-up [5,6]. Given that T2D remission or improvement is a major benefit of bariatric surgery, the ability to preoperatively predict T2D prognosis is crucial for patients and healthcare providers.

Interestingly, a number of studies have highlighted that the circulating concentrations of branched-chain amino acids (BCAAs) and aromatic amino acids, including leucine, isoleucine, valine, phenylalanine, tyrosine, and tryptophan, are closely correlated to insulin resistance and future diabetes [7,8,9,10]. Tryptophan derivatives, especially those from the kynurenine pathway (KynP) and serotonin pathway (SerP), are also highly associated with glucose homeostasis and energy expenditure [11,12,13,14]. Metabolites from the tyrosine pathway (TyrP), such as L-dihydroxyphenylalanine (L-DOPA) and dopamine, have been also suggested to contribute to the interplay between insulin signaling and glucose homeostasis [15,16]. Nonetheless, whether these amino acid metabolites (AAMs) mediate or predict the prognosis of T2D after bariatric surgery remains unclear.

The Korean Obesity Surgical Treatment Study (KOBESS) is based on a nationwide prospective multicenter cohort, the results of which led to the coverage of bariatric surgery by the National Health Insurance for the first time in Korea [17]. In this preplanned substudy of the KOBESS trial conducted to elucidate the role of AAMs as potential predictors of T2D prognosis after bariatric surgery, we investigated whether preoperative AAMs could predict T2D remission after bariatric surgery and compared the prognostic performance of AAMs with that of existing prediction models.

## 2. Methods

### 2.1. Study Participants

This substudy included 24 individuals with type 2 diabetes who participated in the main KOBESS trial (Supplemental Figure S1). The rationale and design of the KOBESS has been previously reported [17]. It was a prospective, multicenter, single-arm, longitudinal study in which 100 patients (including 54 patients with T2D) were enrolled to assess the effect of bariatric surgery on obese patients in Korea. Patients who provided written informed consent were screened for study eligibility and underwent physical and laboratory evaluations to confirm their eligibility (Institutional Review Board approval number: 2019AN0294).

### 2.2. Surgical Procedures

Study participants selected surgical procedures among sleeve gastrectomy (SG) and Roux-en-Y gastric bypass (RYGB) after being fully informed as to the strengths and weaknesses of both. For SG, a sleeve was fashioned starting 4–6 cm proximal to the pylorus using serial applications of linear staplers over a 36–40 Fr orogastric bougie. The last firing was 1–2 cm away from the angle of His. For RYGB, a lesser curve-based gastric pouch (approximately 30 mL in volume) was created using linear staplers. The length of the Roux and biliopancreatic limbs was 100 cm.

### 2.3. Management of Nutrition and Blood Glucose

After study enrollment, all patients were provided with American Diabetes Association dietary and lifestyle recommendations to optimize their glucose control. A bariatric physician and registered dietitian provided patient education and nutrition guidelines, including dietary principles such as carbohydrate counting, advice to engage in regular aerobic exercise (if medically fit to do so according to the physician providing their medical care), technical and interpretive skills of blood glucose monitoring, and education about managing hypoglycemia. All patients started guideline-based micronutrient supplementation after study enrollment and baseline assessment [18]. During the postoperative period, patients received education about a protocol-derived staged meal progression. A protein intake of 50 g/day and up to 1.5 g/kg ideal body weight per day was recommended. All patients were followed up every 4 to 12 weeks via outpatient clinic visit to check on glucose control and ensure compliance with the nutritional recommendations.

### 2.4. Measurements of Serum AAMs

Serum samples were collected before and at 3 and 12 months after bariatric surgery. Preoperative sampling was performed before the patients started the calorie-restricted diet and micronutrient supplementation. Patients fasted for 8 hours before the sampling. AAM profiling was performed using liquid chromatography–mass spectrometry. We selected 16 diabetes-related AAMs such as BCAAs (leucine, isoleucine, and valine), AAAs (phenylalanine, tyrosine, and tryptophan), KynP metabolites (kynurenine, anthranilic acid (AA), 3-hydroxykynurenine (3-HK), 3-hydroxyanthranilic acid (3-HAA), kynurenic acid (KA), xanthurenic acid (XA)), SerP metabolites (5-hydroxytryptophan (5-HTP), serotonin, 5-hydroxyindoleacetic acid (5-HIAA)), and TyrP metabolites (L-dihydroxyphenylalanine (L-DOPA)) based on previous studies on AAMs and glucose homeostasis or T2D (Supplemental Figure S2). The detailed protocol for measuring serum metabolites is presented in Supplemental Tables S1 and S2.

### 2.5. Outcome Measures

We assessed whether serum AAMs could predict T2D remission 12 months after bariatric surgery. T2D remission was defined as a normal glucose level (glycated hemoglobin < 6%, fasting plasma glucose < 100 mg/dL) in the absence of antidiabetic medications at 12 months postoperatively. Non-remission was defined when the criteria for remission were not met. In addition, the prognostic performances of AAMs were compared with scores from the following three prediction models: the ABCD [19], DiaRem [20], and IMS [21] models. Based on a systematic review of the relevant literature, we selected the prediction models that (1) provided scores implying T2D prognosis after bariatric surgery and (2) underwent external validation.

### 2.6. Statistical Analysis

Summary data are presented as percentages for categorical variables and as means with standard deviations (SDs) for continuous variables. Patients’ characteristics were compared between the remission and non-remission groups using the Student’s *t*-test or Mann–Whitney test for continuous variables and the Pearson chi square test or Fisher’s exact test for categorical variables. Areas under receiver operating characteristic curves (AUROCs) were calculated to analyze the performances of the individual AAMs in predicting T2D remission at 12 months postoperatively. Based on the AUROC values, we selected the superior prognostic metabolites, defined as AUROC ≥ 0.80. Statistical analyses were performed using Stata12 (Stata Corp., College Station, TX, USA), and two-sided values of *p* < 0.05 were considered statistically significant.

## 3. Results

### 3.1. Patients’ Baseline Characteristics

The mean age of the study participants was 45.4 years (SD, 10.2 years), and 17 (70.8%) participants were women (Table 1). Among the participants, 14 (58.3%) experienced T2D remission and 10 (41.7%) did not experience T2D remission at 12 months postoperatively. Baseline BMIs were 39.6 kg/m^2^ (SD, 7.9 kg/m^2^) in the remission group and 33.9 kg/m^2^ (SD, 4.5 kg/m^2^) in the non-remission group, respectively. Among the participants, 2 (14.3%) in the remission group and 3 (30.0%) in the non-remission group used insulin for glycemic control preoperatively; 9 (64.3%) in the remission group and 5 (50.0%) in the non-remission group received SG, and the rest of patients received RYGB. The remission and non-remission groups were similar for all observed characteristics, except for age (remission group, 41.0 years (SD, 8.7 years); non-remission group, 54.8 years (SD, 8.0 years); *p* = 0.004) and T2D duration (remission group, 2.2 years (SD, 1.4 years); non-remission group, 8.9 years (SD, 8.6 years); *p* = 0.009). Scores calculated by the three existing prediction models showed significant differences between groups, indicating a higher probability of glycemic control in the remission group.

### 3.2. Patients’ Characteristics at 3 and 12 Months Postoperatively

While the levels of glycated hemoglobin and fasting plasma glucose (FPG) at 3 and 12 months postoperatively were significantly different (*p* value for 3 months, <0.001 (glycated hemoglobin) and <0.001 (FPG); *p* value for 12 months, <0.001 (glycated hemoglobin) and <0.001 (FPG)) between the remission and non-remission groups, both groups were similar in terms of all other observed characteristics at baseline and 3 and 12 months postoperatively (Table 2). The mean glycated hemoglobin levels at baseline and 12 months postoperatively were 7.2% (SD, 2.0%) and 5.4% (SD, 0.2%) in the remission group and 8.9% (SD, 1.1%) and 7.6% (SD, 1.0%) in the non-remission group, respectively. Patients in the remission group presented 62.9% excess weight loss (EWL) (SD, 31.5%) and 90.6% EWL (SD, 38.3%) at 3 and 12 months, respectively, while patients in the non-remission group presented 59.4% EWL (SD, 24.7%) and 68.8% EWL (SD, 32.4%) at 3 and 12 months, respectively. There were no significant differences in blood pressure (systolic and diastolic), HDL cholesterol level, or triglyceride level between the remission and non-remission groups at baseline and 3 and 12 months postoperatively.

### 3.3. Prediction of T2D Remission after Bariatric Surgery

**Figure 1:**
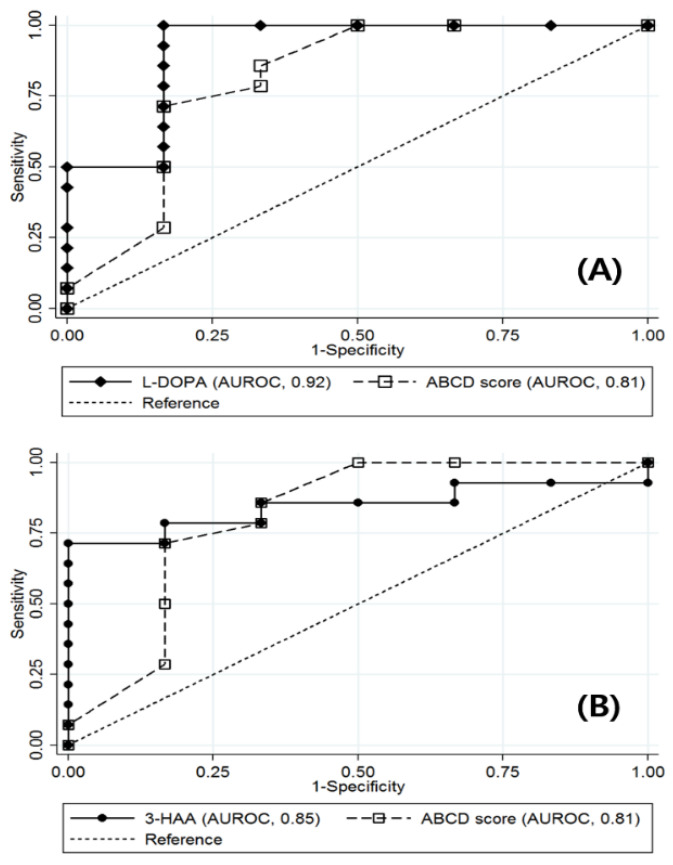
Prognostic performance of L-dihydroxyphenylalanine (L-DOPA), 3-hydroxyanthranilic acid (3-HAA), and the ABCD prediction model. Using 16 diabetes-related amino acid metabolites, receiver operating characteristic (ROC) curves were generated. Among the test serum metabolites, L-DOPA and 3-HAA showed superior prognostic performances with the best discrimination ability in the prediction of type 2 diabetes (T2D) remission 12 months after bariatric surgery. Figure (**A**,**B**) represent the different prognostic outcomes of L-DOPA and 3-HAA, respectively. AUROC, area under receiver operating characteristic curve.

Levels of baseline AAMs were presented in Supplemental Table S3. No significant differences in AAMs were identified with respect to hypertension and dyslipidemia. Based on the receiver operating characteristic (ROC) curves of AAMs, L-DOPA (AUROC, 0.92; 95% CI, 0.75 to 1.00) and 3-HAA (AUROC, 0.85; 95% CI, 0.67 to 1.00) at baseline showed a superior performance in the prediction of T2D remission 12 months after bariatric surgery (Figure 1) (Supplemental Table S4). The ABCD model (AUROC, 0.81; 95% CI, 0.54 to 1.00) showed the highest AUROC among the three existing prediction models, which was lower than those of L-DOPA and 3-HAA (Supplemental Table S5). Given that we found significant differences in age and diabetes duration between the remission and non-remission groups (Table 1), additional analyses were performed on the subgroups therein. The prognostic performance of 3-HAA and L-DOPA for T2D remission was represented by an AUROC ≥ 0.75 (Supplemental Table S6).

### 3.4. Postoperative Changes in L-DOPA and 3-HAA

**Figure 2:**
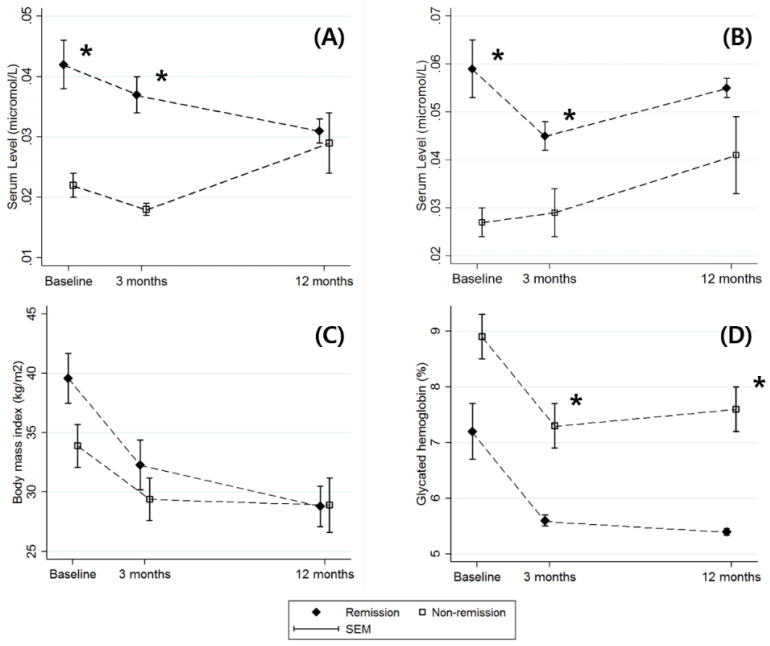
Longitudinal changes of four parameters after bariatric surgery. Figure (**A**–**D**) represent the postoperative changes in L-dihydroxyphenylalanine (L-DOPA), 3-hydroxyanthranilic acid (3-HAA), body mass index, and glycated hemoglobin, respectively. Measurements were obtained at baseline and 3 and 12 months postoperatively. While the serum levels of L-DOPA and 3-HAA at baseline and 3 months were higher in the type 2 diabetes (T2D) remission group, no significant differences between the remission and non-remission groups were identified 12 months postoperatively. * represent *p* < 0.05 in the comparison of the remission and non-remission groups.

**Figure 3:**
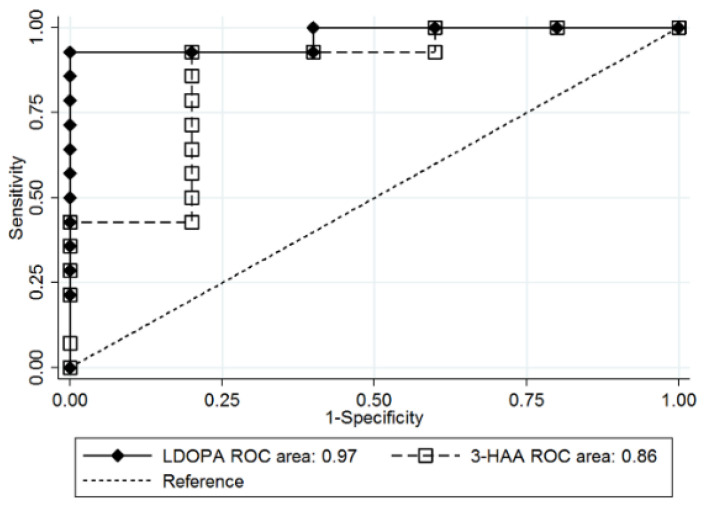
Prognostic performance of L-dihydroxyphenylalanine (L-DOPA) and 3-hydroxyanthranilic acid (3-HAA) measured at 3 months after bariatric surgery. Using L-DOPA and 3-HAA measured at 3 months after surgery, receiver operating characteristic (ROC) curves were generated. The superior prognostic performance of L-DOPA and 3-HAA continued until 3 months postoperatively, and they showed the best discrimination ability in predicting type 2 diabetes (T2D) remission 12 months after bariatric surgery.

Serum levels of L-DOPA and 3-HAA at 3 and 12 months were measured to characterize the postoperative changes between groups (Figure 2) (Supplemental Table S7). The remission group showed higher levels of L-DOPA (remission group, 0.042 μmol/L (SE, 0.004 μmol/L); non-remission group, 0.022 μmol/L (SE, 0.002 μmol/L); *p* = 0.014) and 3-HAA (remission group, 0.059 μmol/L (SE, 0.006 μmol/L); non-remission group, 0.027 μmol/L (SE, 0.003 μmol/L); *p* = 0.005) at baseline. The statistical significance of the higher baseline levels of L-DOPA and 3-HAA was sustained up to 3 months postoperatively (*p* values for 3 months, 0.004 (L-DOPA) and 0.303 (3-HAA)). The superior prognostic performance of baseline L-DOPA and 3-HAA in the prediction of T2D remission was also sustained up to 3 months postoperatively (L-DOPA at 3 months (AUROC, 0.97; 95% CI, 0.91 to 1.00); 3-HAA at 3 months (AUROC, 0.86; 95% CI, 0.63 to 1.00)) (Figure 3). At 12 months, no differences in the levels of L-DOPA and 3-HAA were observed between the groups.

## 4. Discussion

We showed that the profiles of diabetes-related AAMs before bariatric surgery could predict T2D remission 12 months postoperatively. In particular, serum levels of L-DOPA and 3-HAA showed superior prognostic performance with the best discrimination ability at 12 months postoperatively compared with the existing prediction models. We also showed that the superior prognostic performance of L-DOPA and 3-HAA continued for up to 3 months postoperatively.

Our results underscore the importance of KynP metabolites for predicting T2D prognosis after bariatric surgery and must be considered in the context of previous studies suggesting that KynP metabolites were potential biological mediators in T2D pathogenesis [7,9]. The majority of free tryptophan in humans is metabolized via KynP [22], which shifts in favor of the breakdown of tryptophan into downstream products in inflammatory states such as diabetes or obesity [23,24]. Consequently, levels of downstream metabolites such as 3-HAA [25], kynurenine [13], XA [26], and 3-HK [27] are higher in T2D patients than in non-diabetics. In particular, XA was found to contribute to diabetes development by chelating complexes with insulin and exerting pathological apoptosis effects on pancreatic beta cells; furthermore, 3-HAA plays a key role in anti-inflammation and neuroprotection, demonstrating strong antioxidative properties [28].

In patients who underwent bariatric surgery, plasma levels of KynP metabolites decreased 1 year after surgery along with metabolic improvements, implying that KynP metabolites should be assessed as potential biomarkers of metabolic improvement in bariatric patients [27]. According to our results, the baseline level of 3-HAA among the KynP metabolites showed superior performance in predicting T2D remission 1 year after bariatric surgery. Given that no significant differences in the 3-HAA level between the remission and non-remission groups were observed at 12 months postoperatively (Figure 2), our results imply that lower levels of 3-HAA may represent an early manifestation of postoperative T2D remission. Our results are the first to suggest that KynP metabolites predict T2D remission after bariatric surgery and are in line with those of recent studies indicating that KynP metabolites are biological mediators in T2D pathophysiology.

Our findings highlighting the TyrP are noteworthy in the context of experimental and clinical data that suggest that TyrP metabolites may contribute to energy expenditure and glucose homeostasis. Dopamine is synthesized in the beta-cells from circulating L-DOPA, and exogenous dopamine inhibits insulin secretion from pancreatic beta-cells [29,30,31]. In this negative feedback loop, dopamine serves as an autocrine signal that is co-secreted with insulin and causes a tonic inhibition on glucose-stimulated insulin secretion [29,30,31]. Beta-cells co-secrete dopamine as well as insulin in response to glucose stimulus and express dopamine 2-like receptors (D2R), which are responsible for the import of L-DOPA [32]. Experiments with dopamine antagonists suggest that the co-secreted dopamine binds to D2R to downregulate beta-cell insulin secretion [32].

In the present study, the T2D remission group showed higher levels of serum L-DOPA at baseline and 3 months postoperatively than the non-remission group. Recently, it has been suggested that dopamine and glucagon-like peptide-1 exert opposing effects on the glucose-stimulated insulin secretion regulatory system and the anti-incretin effect of dopamine have been indicated as a potential mechanism of diabetes remission after bariatric surgery [16,33]. Given that the proximal gastrointestinal tract is the major source of peripheral circulating dopamine and L-DOPA [34], bariatric surgeries such as SG and RYGB might reduce the secretion and anti-incretin effect of dopamine and L-DOPA. This hypothesis regarding the decrease in dopaminergic action after bariatric surgery is in line with our findings; while the serum level of L-DOPA decreased continuously until 12 months after surgery in the remission group, no significant difference between baseline and 12 months was identified in the non-remission group (Figure 2).

For the preoperative prediction of T2D remission, several prediction models have been proposed. However, these prediction models have been challenged by insufficient validation studies and discordance between models [35]. We calculated the AUROCs of the three existing prediction models (the ABCD, DiaRem, and IMS models) and clinical parameters, according to existing prediction models for the prediction of T2D remission after bariatric surgery (Supplemental Table S5). However, the prognostic performances of the AAMs (especially L-DOPA and 3-HAA) were superior to those of the existing prediction models and other clinical parameters. Hence, AAMs should be further evaluated and considered as potential biomarkers for T2D remission.

This study had some limitations. First, our results did not exclude the possibility that other AAMs may also predict T2D remission after bariatric surgery. Further analysis of downstream KynP metabolites, such as quinolinic acid and nicotinamide adenine dinucleotide, and BCAA metabolites, such as alanine, glutamine, and glutamate, is necessary. Second, the postoperative individual diet pattern may affect the prognosis of T2D postoperatively. In animal studies, a low-BCAA diet improved glycemic control independent of energy balance [36], whereas leucine enriched the diet and improved glucose homeostasis [37]. Further studies to investigate the effect of postoperative diet pattern on the predictive performance of AAMs in bariatric patients are warranted. Third, the subjects were not randomized to the surgical procedures. In light of the high prevalence of gastric cancer in Korea, allocation to RYGB could raise ethical issues, because postoperative esophagogastroduodenoscopy for gastric cancer screening is not allowed in patients undergoing RYGB [17]. Although no significant difference in the surgical methods between the remission and non-remission groups was identified, further investigation of the effect of surgical methods on the prognostic performance of AAMs is warranted. Fourth, our study was conducted on a small number of Asian patients with relatively low BMIs. Caution is advised in applying our results to patients of other ethnicities, or patients with higher BMIs. Our results should be further validated in studies with more patients.

In conclusion, our data provide new insights into preoperative AAMs and the prediction of T2D remission after bariatric surgery. In our comprehensive metabolomics study targeting diabetes-related AAMs, preoperative levels of L-DOPA and 3-HAA more effectively predicted T2D remission 1 year after bariatric surgery compared with the existing prediction models. In addition, the predictive performance of these two metabolites persisted until 3 months postoperatively. The higher levels of L-DOPA and 3-HAA in the T2D remission group have significant clinical implications for the further development and application of surgical indications for the treatment of obesity and T2D.

## Figures and Tables

**Table 1:** Baseline characteristics.

Variables	Diabetes Status 1 Year after Bariatric Surgery		*p* Value
	Remission (*n* = 14)	Non-Remission (*n* = 10)	
Age, y	41.0 ± 8.7	54.8 ± 8.0	0.004
Female sex, no. (%)	12 (85.7)	5 (50.0)	0.058
Body mass index, kg/m^2^	39.6 ± 7.9	33.9 ± 4.5	0.116
Body weight, kg	106.1 ± 31.2	89.7 ± 18.4	0.251
Waist circumference, cm	120.5 ± 19.9	112.1 ± 9.7	0.347
Waist-to-hip ratio	0.97 ± 0.05	1.03 ± 0.01	0.017
Duration of diabetes, y	2.2 ± 1.4	8.9 ± 8.6	0.009
Use of insulin, no. (%)	2 (14.3)	3 (30.0)	0.350
Current smoker, no. (%)	3 (21.4)	2 (20.0)	0.932
Hypertension, no. (%)	5 (35.7)	5 (50.0)	0.484
Dyslipidemia, no. (%)	9 (64.2)	6 (60.0)	0.831
Surgical methods (RYGB/SG), no. (%)	5/9 (35.7/64.3)	5/5 (50.0/50.0)	0.484
ABCD score	6.2 ± 2.0	3.3 ± 2.7	0.015
DiaRem score	4.7 ± 5.4	12.0 ± 5.3	0.013
IMS score	39.3 ± 22.9	88.2 ± 30.6	0.001

Plus–minus values are mean ± standard deviation. Remission was defined as normal glucose level (glycated hemoglobin < 6%, fasting plasma glucose < 100 mg/dL) in the absence of antidiabetic medications at 1 year postoperatively. Non-remission was defined when the criteria for remission were not met. Body mass index is the weight in kilograms divided by the square of the height in meters. Refer to the Methods section for details of the ABCD, DiaRem, and IMS scores. Abbreviations: RYGB, Roux-en-Y gastric bypass; SG, sleeve gastrectomy.

**Table 2:** Average values and percentage changes at 3 and 12 months after bariatric surgery.

Variables	Remission			Non-Remission			*p* Value for Baseline	*p* Value for 3 Months	*p* Value for 12 Months
	Baseline (*n* = 14)	3 Months (*n* = 14)	12 Months (*n* = 14)	Baseline (*n* = 10)	3 Months (*n* = 10)	12 Months (*n* = 10)			
Glycated hemoglobin, %	7.2 ± 2.0	5.6 ± 0.3	5.4 ± 0.2	8.9 ± 1.1	7.3 ± 1.1	7.6 ± 1.0	0.067	<0.001	<0.001
Fasting plasma glucose, mg/dL	151.1 ± 65.3	98.3 ± 9.8	97.2 ± 7.8	146.1 ± 51.9	129 ± 22.9	131 ± 26.6	0.871	<0.001	<0.001
Body mass index, kg/m^2^	39.6 ± 7.9	32.3 ± 7.7	28.8 ± 6.6	33.9 ± 4.5	29.4 ± 4.5	28.9 ± 5.6	0.116	0.409	0.974
Body weight, kg	106.1 ± 31.2	87.7 ± 29.8	77.0 ± 24.9	89.7 ± 18.4	77.9 ± 17.6	76.7 ± 20.1	0.251	0.471	0.977
% Excess weight loss	-	62.9 ± 31.5	90.6 ± 38.3	-	59.4 ± 24.7	68.8 ± 32.4	-	0.818	0.240
% Weight loss	-	18.2 ± 4.1	27.6 ± 5.2	-	13.4 ± 2.7	15.1 ± 5.8	-	0.019	<0.001
Waist circumference, cm	120.5 ± 19.9	104.6 ± 21.0	93.8 ± 16.5	112.1 ± 9.7	98.4 ± 12.4	98.1 ± 12.5	0.347	0.515	0.573
Systolic blood pressure, mmHg	135.7 ± 9.8	125.1 ± 12.1	120.1 ± 13.1	136.8 ± 8.4	124.1 ± 11.5	130 ± 10.2	0.812	0.870	0.120
Diastolic blood pressure, mmHg	84.5 ± 9.8	77.6 ± 10.9	74.5 ± 9.1	85.0 ± 4.9	73.6 ± 12.7	77.1 ± 11.40	0.908	0.496	0.585
High-density lipoprotein cholesterol, mg/dL	49.5 ± 9.8	48.6 ± 10.7	60.1 ± 12.2	46.6 ± 8.5	46.1 ± 13.1	52.5 ± 11.2	0.549	0.671	0.208
Triglycerides, mg/dL	204.4 ± 232.3	110.6 ± 38.7	94.5 ± 36.0	174.6 ± 140.6	149.5 ± 88.6	139 ± 77.5	0.776	0.193	0.091

Plus–minus values are mean ± standard deviation. Body mass index is the weight in kilograms divided by the square of the height in meters. % Excess weight loss was calculated by dividing the number of kilograms lost by the number of kilograms of the patient’s excess body weight. % Weight loss was calculated by dividing the number of kilograms by the number of kilograms of the patient’s baseline body weight.

## Supplementary Materials

The following are available online at https://www.mdpi.com/2077-0383/9/12/3897/s1, Table S1: Protocol for measurement of serum amino acids metabolites; Table S2: Chromatographic retention time (RT), selected MRM parameters, DP, EP, CE, and CXP for each analyte measured; Table S3: Levels of baseline amino acid metabolites according to comorbidities; Table S4: Prognostic performance of amino acid metabolites to predict diabetes remission 12 months after bariatric surgery; Table S5: Prognostic performance of existing prediction models and clinical parameters to predict diabetes remission 12 months after bariatric surgery; Table S6, Subgroup analyses; Table S7, Longitudinal changes of serum metabolites after bariatric surgery; Figure S1: Study participants; Figure S2: Diabetes-related amino acid metabolites.
