# Supplementary material for: Metabolomic Profiles Predict Diabetes Remission after Bariatric Surgery
Source: J Clin Med. 2020 Dec 1;9(12):3897. doi: 10.3390/jcm9123897 (PMC7760750; doi:10.3390/jcm9123897)
Supplement: Supplementary file 1 [file jcm-09-03897-s001.pdf]

Supplemental files:

Table S1. Protocol for measurement of serum amino acids metabolites.

1. Amino acids and their metabolites were quantified using liquid chromatography-mass spectrometry (LC-MS) based on previous publication methods[1,2]. Briefly, 10 µL of ^{13}C-tryptophan (5 µM), as the internal standard (IS), was added to 100 µL of serum and standard solution in 4% bovine serum albumin solution and diluted with 20 µL of water containing 0.1% formic acid (v/v). Subsequently, 400 µL of cold acetonitrile for protein precipitation was added, vortexed, and centrifuged at 10,000 x g at 4 °C for 10 min. Next, 400 µl of the supernatant was dried using a speed vacuum evaporator and reconstituted in 0.1% formic acid in water/acetonitrile (9/1, v/v) solution. Then, 10 µL of the samples was injected into an HPLC-MS/MS system (Agilent 1200 HPLC equipped with AB SCIex 3200 Mass analyzer) equipped with the Waters Atlantis T3 (4.6×150 mm i.d., 3 µm) column at 30 °C.
2. Ions of each analyzed compound, except the IS, were detected in a positive ionization mode using the multiple reaction monitoring mode. Liquid chromatographic separation was performed with mobile phases A (0.1% formic acid in water, v/v) and B (0.1% formic acid in acetonitrile, v/v) at 0.5 mL/min flow rate under the following conditions: 5% for the first gradient starting at 5% B to 40% B in 6 min, to 90% B in 5 min, staying at 90% B for 5 min, then to 5% B in 1 min. The column equilibration was performed for 8 min before each analysis under the 5% B condition.
3. In terms of the MS conditions, the ion source temperature was set to 600 °C. The curtain and nebulizer gas were set at 20 psi and 50 psi, respectively. The MS capillary voltage was 4.5 kV for the positive mode or -4.5 kV for the negative mode. The acquired data were analyzed using Analyst software (v1.6.3, SCIex, USA).

[1] Zhu W, Stevens AP, Dettmer K, et al. Quantitative profiling of tryptophan metabolites in serum, urine, and cell culture supernatants by liquid chromatography-tandem mass spectrometry. Anal Bioanal Chem 2011;401:3249-3261. [2] Choi JM, Park WS, Song KY, et al. Development of simultaneous analysis of tryptophan metabolites in serum and gastric juice - an investigation towards establishing a biomarker test for gastric cancer diagnosis. Biomed Chromatogr 2016;30:1963-1974.

Table S2. Chromatographic retention time (RT), selected MRM parameters, DP, EP, CE, and CXP for each analyte measured.

Compounds	Exact Mass	RT	Ion mode	(MH+) or (M-H)-	Product Ion Mass	DP	EP	CE	CXP
Leucine ($C_6H_{13}NO_2$)	131.095	6.5	Pos	132.1	43.2	21	7.5	31.	10
					86.1	21	7.5	31.0	10
Isoleucine ($C_6H_{13}NO_2$)	131.095	6.2	Pos	132.0	86.1	26	5.5	21.0	10
Valine ($C_5H_{11}NO_2$)	117.0789	4.1	Pos	118.1	72.1	26	5.0	19.0	10
Phenylalanine ($C_9H_{11}NO_2$)	165.079	7.3	Pos	166.2	103.2	21	3.0	33.0	10
					120.2	21	3.0	19.0	10
Tyrosine ($C_9H_{11}NO_3$)	181.074	6.3	Pos	181.9	91.2	26	6.0	35.0	14
					136.1	26	5.5	35.0	14
Tryptophan ($C_{11}H_{12}N_2O_2$)	204.09	8.1	Pos	205.0	117.9	26	6.0	33.0	12
					158.8	26	6.0	33.0	12
Kynurenine ($C_{10}H_{12}N_2O_3$)	208.0848	7.4	Pos	209.1	94.0	26	5.0	19.0	10
					192.200	26	5.0	13.0	10.0
Kynurenic acid ($C_{10}H_7NO_3$)	189.043	9.2	Pos	189.900	116.000	31	3.0	39.0	12.0
					143.900	21	3.0	39.0	12.0
Anthranilic acid ($C_7H_7NO_2$)	137.048	11.4	Pos	138.106	92.100	16	3.0	27.0	14.0
					119.900	16	3.0	15.0	14.0
Xanthurenic acid ($C_{10}H_7NO_4$)	205.0375	8.1	Pos	206.071	132.200	51	10.5	41.0	12.0
					159.900	51	10.5	23.0	12.0
3-Hydroxykynurenine ($C_{10}H_{12}N_2O_4$)	224.0797	6.2	Pos	225.061	110.100	26	8.5	23.0	20.0
					208.100	26	8.5	13.0	20.0
3-Hydroxyanthranilic acid ($C_7H_7NO_3$)	153.043	9.0	Pos	154.000	108.100	26	2.5	25.0	10.0
					136.200	21	2.0	25.0	10.0
5-hydroxyindoleacetic acid ($C_{10}H_9NO_3$)	191.058	9.6	Pos	192.100	146.100	26.0	5.5	29.0	10.0
5-Hydroxytryptophan ($C_{11}H_{12}N_2O_3$)	220.084	6.9	Pos	221.100	175.000	26.0	5.5	23.0	12.0
L-Dihydroxyphenylalanine ($C_9H_{11}NO_4$)	197.069	5.0	Pos	197.934	107.200	36.0	4.0	29.0	14.0
					152.000	36.0	4.0	21.0	14.0
	215.09	8.3	Pos	216.100	126.100	26.0	5.5	33.0	12.0

IS(^{13}C-Tryptophan) ($C_{11}H_{12}N_2O_2$)	Neg	214.000	124.100	-50.0	-10.0	-24.0	-12.0

MRM: multiple reaction mode, DP: declustering potential, EP: entrance potential, CE: collision energy, CXP: collision cell exit potential, IS: internal standard, (MH+): positive ion mode, (M-H)-: negative ion mode.

Table S3. Levels of baseline amino acid metabolites according to comorbidities.

**Amino acid metabolites (abbreviations), (μmol/L)**	**Overall**	**Hypertension**		*P* value	Dyslipidemia		*P* value
		**Yes**	No		Yes	No	
**BCAAs**							
Leucine (Leu)	144.1 (4.3)	144.0 (7.7)	144.3 (4.3)	0.979	145.6 (5.4)	139.2 (4.9)	0.545
Isoleucine (Iso)	96.4 (3.8)	96.9 (6.1)	96.0 (4.9)	0.906	98.3 (4.5)	90.2 (7.2)	0.390
Valine (Val)	216.4 (7.8)	214.8 (13.3)	218.0 (9.0)	0.842	217.3 (9.6)	213.5 (13.2)	0.844
Sum of BCAAs	457.1 (14.3)	455.8 (24.7)	458.4 (15.6)	0.932	461.3 (17.5)	442.9 (21.5)	0.602
AAAs							
Phenylalanine (Phe)	106.2 (4.0)	101.3 (6.8)	111.1 (4.2)	0.237	104.1 (4.9)	113.4 (5.1)	0.346
Tyrosine (Tyr)	78.9 (2.9)	74.0 (4.1)	83.8 (3.8)	0.098	78.7 (3.3)	79.7 (6.8)	0.887
Tryptophan (Trp)	81.9 (2.7)	81.1 (4.6)	82.7 (3.3)	0.781	81.1 (3.3)	84.6 (5.2)	0.608
Sum of BCAAs and AAAs	724.3 (19.9)	712.4 (34.6)	736.2 (20.8)	0.564	725.3 (24.6)	720.8 (30.2)	0.927
Metabolites from the kynurenine pathway							
Kynurenine (Kyn)	3.018 (0.204)	2.967 (0.333)	3.069 (0.251)	0.808	3.029 (0.253)	2.982 (0.295)	0.927
Anthranilic acid (AA)	0.054 (0.003)	0.052 (0.005)	0.055 (0.004)	0.697	0.053 (0.003)	0.056 (0.008)	0.758
3-hydroxykynurenine (3-HK)	0.085 (0.007)	0.085 (0.013)	0.084 (0.009)	0.980	0.086 (0.009)	0.078 (0.014)	0.672
3-hydroxyanthranilic acid (3-HAA)	0.048 (0.005)	0.046 (0.006)	0.051 (0.008)	0.613	0.047 (0.005)	0.054 (0.014)	0.568
Kynurenic acid (KA)	0.069 (0.007)	0.073 (0.013)	0.064 (0.006)	0.546	0.069 (0.009)	0.068 (0.010)	0.948
Xanthurenic acid (XA)	0.035 (0.003)	0.038 (0.006)	0.031 (0.003)	0.325	0.035 (0.004)	0.033 (0.003)	0.829
Metabolites from the serotonin pathway							
5-hydroxytryptophan (5-HTP)	1.605 (0.110)	1.575 (0.179)	1.635 (0.138)	0.796	1.617 (0.140)	1.566 (0.129)	0.851
Serotonin (Ser)	0.499 (0.046)	0.525 (0.085)	0.473 (0.043)	0.595	0.454 (0.052)	0.654 (0.071)	0.073
5-hydroxyindoleacetic acid (5-HIAA)	0.057 (0.004)	0.056 (0.004)	0.057 (0.007)	0.613	0.059 (0.005)	0.048 (0.005)	0.568
Metabolites from the tyrosine pathway							
L-dihydroxyphenylalanine (L-DOPA)	0.034 (0.003)	0.033 (0.006)	0.036 (0.005)	0.702	0.035 (0.004)	0.031 (0.003)	0.675

Numbers represent mean (standard error).

Table S4. Prognostic performance of amino acid metabolites to predict diabetes remission 12 months after bariatric surgery.

**Amino acid metabolites (abbreviations)**	**Area under ROC curve (SE)**	**95% CI**
BCAAs		
Leucine (Leu)	0.60 (0.17)	0.28 to 0.92
Isoleucine (Iso)	0.41 (0.17)	0.08 to 0.75
Valine (Val)	0.65 (0.14)	0.38 to 0.93
Sum of BCAAs	0.64 (0.16)	0.33 to 0.95
AAAs		
Phenylalanine (Phe)	0.64 (0.18)	0.28 to 0.99

Tyrosine (Tyr)	0.77 (0.14)	0.51 to 1.00
Tryptophan (Trp)	0.76 (0.13)	0.51 to 1.00
Sum of BCAAs and AAAs	0.68 (0.14)	0.40 to 0.96
Metabolites from the kynurenine pathway		
Kynurenine (Kyn)	0.59 (0.17)	0.26 to 0.91
Anthranilic acid (AA)	0.49 (0.17)	0.16 to 0.81
3-hydroxykynurenine (3-HK)	0.76 (0.11)	0.54 to 0.99
3-hydroxyanthranilic acid (3-HAA)	0.85 (0.09)	0.67 to 1.00
Kynurenic acid (KA)	0.57 (0.16)	0.27 to 0.88
Xanthurenic acid (XA)	0.60 (0.16)	0.28 to 0.91
Metabolites from the serotonin pathway		
5-hydroxytryptophan (5-HTP)	0.63 (0.18)	0.28 to 0.98
Serotonin (Ser)	0.70 (0.15)	0.41 to 1.00
5-hydroxyindoleacetic acid (5-HIAA)	0.73 (0.13)	0.47 to 0.98
Metabolites from the tyrosine pathway		
L-dihydroxyphenylalanine (L-DOPA)	0.92 (0.09)	0.75 to 1.00

Abbreviations: ROC, receiver operating characteristic; SE, standard error; CI, confidence interval; BCAA, branched-chain amino acid; AAA, aromatic amino acid.

Table S5. Prognostic performance of existing prediction models and clinical parameters to predict diabetes remission 12 months after bariatric surgery.

**Variables**	**Area under ROC curve (SE)**	**95% CI**
Age	0.14 (0.10)	0.00 to 0.33
Body mass index	0.74 (0.12)	0.49 to 0.98
Waist circumference	0.57 (0.13)	0.31 to 0.83
Waist-to-hip ratio	0.13 (0.08)	0.00 to 0.29
Duration of diabetes	0.27 (0.17)	0.00 to 0.61
Glycated hemoglobin	0.19 (0.11)	0.00 to 0.40
Fasting plasma glucose	0.45 (0.16)	0.14 to 0.77
ABCD score	0.81 (0.14)	0.54 to 1.00
DiaRem score	0.10 (0.07)	0.00 to 0.24
IMS score	0.11 (0.07)	0.00 to 0.25

Abbreviations: ROC, receiver operating characteristic; SE, standard error; CI, confidence interval.

Table S6. Subgroup analyses.

**Subgroups**	**3-hydroxyanthranilic acid (3-HAA)**		**L-dihydroxyphenylalanine (L-DOPA)**	
	**Area under ROC curve (SE)**	**95% CI**	**Area under ROC curve (SE)**	**95% CI**
Age, ≥ 50 years	1.00 (0.00)	1.00 to 1.00	0.80 (0.20)	0.40 to 1.00
Age, < 50 years	0.80 (0.11)	0.59 to 1.00	1.00 (0.00)	1.00 to 1.00
Diabetes duration, ≥ 3 years	0.75 (0.17)	0.40 to 1.00	0.87 (0.13)	0.60 to 1.00
Diabetes duration, < 3 years	0.87 (0.12)	0.63 to 1.00	1.00 (0.00)	1.00 to 1.00

Abbreviations: ROC, receiver operating characteristic; SE, standard error; CI, confidence interval

Table S7. Longitudinal changes of serum metabolites after bariatric surgery.

**Metabolites (μmol/L)**	**Remission**			Non-remission			*P* value for baseline	*P* value for 3 months	*P* value for 12 months
	**Baseline (*n* = 14)**	**3 months (*n* = 14)**	12 months (*n* = 14)	Baseline (*n* = 10)	3 months (*n* = 10)	12 months (*n* = 10)			
**L-DOPA**	**0.042 (0.004)**	**0.037 (0.003)**	0.031 (0.002)	0.022 (0.002)	0.018 (0.001)	0.029 (0.005)	0.014	0.004	0.771
3-HAA	0.059 (0.006)	0.045 (0.003)	0.055 (0.002)	0.027 (0.003)	0.029 (0.005)	0.041 (0.008)	0.005	0.030	0.083

Numbers represent mean (standard error). Abbreviations: L-DOPA, L-dihydroxyphenylalanine; 3-HAA, 3-hydroxyanthranilic acid.

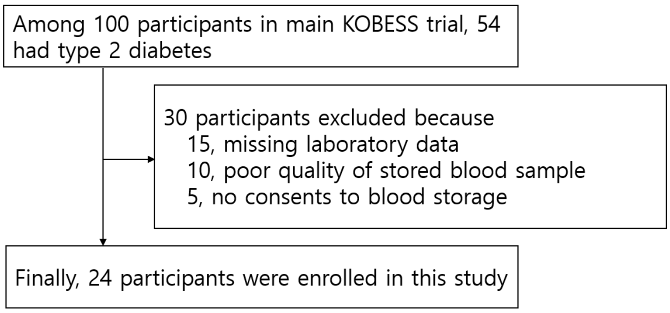

Figure S1. Study participants.

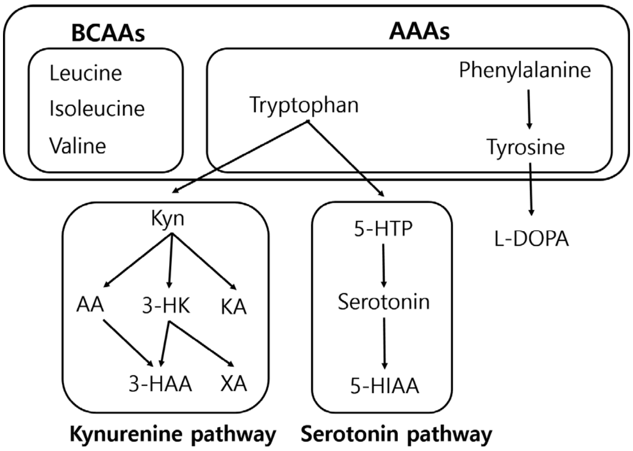

Figure S2. Diabetes-related amino acid metabolites.
